# Association between non-high-density lipoprotein to high-density lipoprotein cholesterol ratio and bowel health in U.S. adults: a cross-sectional study

**DOI:** 10.3389/fphys.2025.1501171

**Published:** 2025-04-24

**Authors:** Wei Liu, Qirui Liu, Cheng Jiao, Jun Guo, Lipu Zhang, Yao Zhang, Guangchao Liu

**Affiliations:** ^1^ Department of General Surgery, Bethune International Peace Hospital, Shijiazhuang, Hebei, China; ^2^ Preparatory Class for Ethnic Minorities, Hebei University, Shijiazhuang, Hebei, China

**Keywords:** non-high-density lipoprotein cholesterol to high-density lipoprotein cholesterol ratio, national health and nutrition examination survey, dyslipidemia, bowel health, chronic constipation, chronic diarrhea

## Abstract

**Background:**

The non-high-density lipoprotein cholesterol to high-density lipoprotein cholesterol ratio (NHHR) is a robust predictor of dyslipidemia and cardiovascular disease, strongly linked to the development of various chronic conditions. However, there is a paucity of evidence exploring the relationship between NHHR and bowel health, particularly chronic diarrhea and constipation.

**Methods:**

This cross-sectional study utilized data from the National Health and Nutrition Survey (NHANES) 2005–2010. Sociodemographic, lifestyle, and health status data were collected alongside blood lipid levels. Weighted multivariate logistic regression models assessed the association between NHHR and bowel health. The restricted cubic spline (RCS) method was used to explore their dose-response relationship. Subgroup analyses and sensitivity analyses were conducted to further validate the robustness of our findings.

**Results:**

In our study of 11,268 participants, a significant positive association was identified between elevated NHHR levels and chronic constipation in women, with the highest quartile showing an adjusted OR of 1.57 (95% CI: 1.21–2.03) compared to the lowest quartile. This association was notably stronger among female smokers. Sensitivity analyses excluding individuals with hypercholesterolemia or inflammatory bowel disease confirmed the robustness of the correlation. No significant associations were found in men.

**Conclusion:**

The study findings provide novel evidence of the relationship between NHHR and bowel health in United States women, particularly chronic constipation. However, the cross-sectional design of the study limits our ability to establish causality. Additionally, reliance on self-reported bowel health data may introduce inaccuracies. Further research is needed to explore the mechanisms underlying this association and the impact of lifestyle factors.

## 1 Introduction

Dyslipidemia, marked by elevated levels of total cholesterol, low-density lipoprotein cholesterol (LDL-C), and triglycerides, and reduced high-density lipoprotein cholesterol (HDL-C) levels, is a major risk factor for ischemic heart disease (IHD), imposing a considerable global health burden (Safiri et al., 2022). The Global Burden of Disease Study has reported an upward trend in plasma LDL-C levels, particularly in conjunction with socio-economic development (GBD 2017 Risk Factor Collaborators, 2018). This trend is exacerbated by shifts in dietary habits and the rise of unhealthy lifestyles, especially in urbanizing regions.

The non-high-density lipoprotein cholesterol (Non-HDL-C) to HDL-C ratio (NHHR) is a robust indicator of blood lipid levels, reflecting the balance between atherogenic and antiatherogenic lipids (Tan et al., 2024). NHHR is a strong predictor of cardiovascular disease and is associated with insulin resistance, metabolic syndrome, and the advancement of some chronic conditions (Tan et al., 2024; Kim et al., 2013). Recent studies have highlighted the potential of NHHR as a biomarker for various metabolic disorders, including type 2 diabetes (Tan et al., 2024), suggesting its broader relevance in understanding the interplay between lipid metabolism and overall health.

Gastrointestinal health is intricately linked to lipid metabolism, crucial for overall health maintenance (Ko et al., 2020). Chronic intestinal dysfunctions, such as diarrhea and constipation, are increasingly prevalent and significantly impair quality of life (Mearin et al., 2016). These conditions are also significantly linked to the development of various chronic diseases such as cardiovascular diseases, diabetes, and certain cancers (Peng et al., 2022).

The intestinal tract’s sensitivity to lipid metabolism dysregulations suggests that lipid balance may significantly impact its function (Rizzetto et al., 2018). Recent research has begun to explore the potential association between NHHR and bowel health. For instance, a cross-sectional study observed a link between higher NHHR and a greater prevalence of periodontitis, which may be connected to intestinal microbiota imbalances (Hou et al., 2024). Given the established role of NHHR as a predictor for type 2 diabetes and its associations with changes in the intestinal microbiota and inflammatory processes (Tan et al., 2024), it is reasonable to hypothesize that NHHR could also influence bowel health. However, the specific relationship between NHHR and bowel health remains underexplored. While some studies have investigated the broader association between lipid profiles and bowel health, the unique predictive value of NHHR has not been adequately addressed (Hou et al., 2024; Jørgensen et al., 2024). This gap in the literature highlights the need for further investigation into NHHR’s role in bowel health.

Given the association of NHHR with chronic diseases and its potential as a biomarker for bowel health, further exploration is warranted. This study aimed to investigate the correlation between NHHR levels and bowel health among the United States population. We hypothesize that elevated NHHR levels may be associated with adverse bowel health outcomes, potentially through mechanisms involving inflammation, oxidative stress, and alterations in the gut microbiome.

## 2 Materials and methods

### 2.1 Data source and study population

**FIGURE 1:**
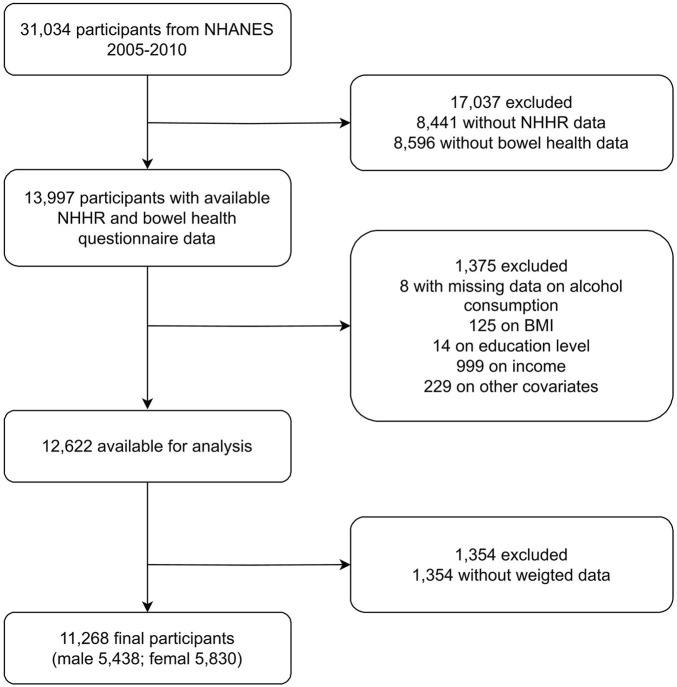
Flowchart of the study.

This cross-sectional analysis leveraged data from National Health and Nutrition Examination Survey (NHANES) from 2005 to 2010, conducted by the Centers for Disease Control and Prevention (CDC). NHANES comprehensively assesses the health status and dietary patterns of noninstitutionalized Americans. Participants were selected via stratified probability sampling and provided informed consent. The study was approved by the National Center for Health Statistics (NCHS) Ethics Review Board, and subsequent analyses were exempt from additional review. NHANES data includes blood lipid levels but not clinical diagnoses of dyslipidemia or hyperlipidemia. Thus, our analysis is based on lipid measurements, not clinical diagnoses. Figure 1 presents the study flowchart, outlining the inclusion and exclusion criteria, and specifies reasons for participant exclusion, including missing data on key variables (NHHR and bowel health) and incomplete covariate information.

### 2.2 Assessment of bowel health

Bowel health was evaluated using the Bristol Stool Form Scale (BSFS) and bowel movement frequency at NHANES’s Mobile Examination Center (MEC). Chronic constipation and diarrhea were diagnosed based on specific BSFS types and bowel movement counts. Participants were identified as having chronic constipation based on a BSFS score of one or two and less than three bowel movements weekly. Chronic diarrhea was identified by a BSFS score of 6 or 7 and more than three movements daily. BSFS scores of 3, 4, 5, and other movements indicated normal bowel function, excluding them from the chronic conditions categories (Peng et al., 2022).

### 2.3 Calculation of NHHR

Blood samples were processed, stored, and shipped to the University of Minnesota, Minneapolis for subsequent analysis. NHHR were calculated from the lipid profiles, applying the formula: NHHR = Non-HDL-C/HDL-C, with Non-HDL-C defined as total cholesterol (TC) minus HDL-C.

### 2.4 Assessment of covariates

The association between NHHR and bowel health was adjusted for demographic factors (age, race/ethnicity, education, marital status, family income, body mass index (BMI)), lifestyle factors (physical activity, cholesterol intake, smoking, alcohol consumption), and health conditions (hypertension, diabetes, malignancy history, etc.). Race categories included non-Hispanic white, non-Hispanic black, Mexican, and other racial/ethnic groups. Education levels were categorized as less than 9 years, 9–11 years, and 12 years or more. Family income levels were stratified into low, moderate, and high (Liu et al., 2022). Smoking status was recorded as never smoker and current smoker (Kim et al., 2022), while alcohol consumption was categorized as current and non-drinkers (Guo et al., 2021). Assessment of physical activity levels was conducted using the Global Physical Activity Questionnaire (GPAT), which included questions on routine and recreational activities, calculated into metabolic equivalents for assessment (Zhao et al., 2020). Dietary cholesterol intake was evaluated through a 24-h dietary recall questionnaire, with the initial interview conducted at the MEC and a follow-up by telephone 3–10 days later, averaging the results of both interviews for accuracy. Information on hypertension, diabetes, inflammatory bowel disease (IBD) and malignancy history was also collected via recall questionnaires.

### 2.5 Statistical analysis

A secondary analysis of NHANES data was performed for this study. Weighted analysis utilized next-day dietary weights (WTDR2D), with sampling weights computed as 1/3 × WTDR2D. Categorical data were presented as unweighted frequencies along with corresponding weighted percentages. Continuous variables were presented as mean ± SD for normally distributed data and median and IQR for skewed data. Statistical comparisons included one-way ANOVA for normal data, the Kruskal–Wallis test for skewed data, and the weighted chi-square test for categorical data to evaluate group differences.

A stepwise multivariate weighted regression model assessed the NHHR-bowel health relationship. Model one adjusted for age, race/ethnicity, education, marital status, family income, and BMI. Model two additionally included physical activity, dietary cholesterol intake, smoking, and alcohol consumption. Model three included additional hypertension, diabetes, and cancer.

To examine trends and nonlinear relationships between NHHR and bowel health, we employed a weighted multiple regression model, incorporating the median of quartile values of NHHR as a continuous variable. We utilized a restricted cubic spline with knots at the 10th, 50th, and 90th percentiles and improved accuracy by excluding the extreme 5% of values. The nonlinearity of the model was evaluated using the likelihood ratio test, comparing it to a model with both linear and cubic spline components.

To further elucidate differences among subgroups, we conducted subgroup analyses and assessed interactions using likelihood ratio tests. To ensure the robustness of our findings, we conducted several sensitivity analyses. First, we excluded patients with hypercholesterolemia (defined as non-HDL-C ≥ 160 mg/dL) and those with inflammatory bowel disease (IBD, including ulcerative colitis and Crohn’s disease). These exclusions were limited by IBD data availability, with only 42 participants out of 11,268 from the 2009–2010 questionnaire included in this analysis. Additionally, we included lipid-lowering medication use as a dichotomous variable in our regression models to account for its potential confounding effects. Furthermore, to address missing covariate data, we employed multiple imputation techniques. Specifically, we used multiple imputation by chained equations (MICE) to generate multiple complete datasets. We performed our primary analyses on each of the imputed datasets and pooled the results to obtain final estimates. The results of these sensitivity analyses help to strengthen the robustness of our conclusions.

Statistical analyses were conducted using R Statistics software (version 4.2.2, R Foundation, http://www.R-project.org) and the Free Statistics analysis platform (version 1.9, Beijing, China, http://www.clinicalscientists.cn/). A *P*-value threshold of 0.05 indicated statistical significance for two-tailed tests.

## 3 Results

### 3.1 Baseline characteristics

The study encompassed 3 year cycles of NHANES, initially involving 31,034 survey respondents, from which 11,268 participants were ultimately included. Among them, 1,103 (9.41%) reported chronic constipation, 981 (7.89%) had chronic diarrhea, and 9,184 (82.70%) exhibited normal bowel health. Detailed baseline characteristics of both excluded and included participants are in Supplementary Table S1. Participants were categorized by NHHR quartiles for baseline characteristics, as shown in Table 1. The mean age of participants was 49.63 ± 17.82 years, with 5,438 males and 5,830 females. Higher NHHR levels were associated with younger men, lower education, lower family income, smoking, high cholesterol diets, and prevalent hypertension and diabetes.

**TABLE 1:** Characteristics of participants in the NHANES 2005–2010 cycles.

Characteristic	Participants^a^					*P*-value
	Total (n = 11,268) NHHR	Q1 (n = 2,774) <2.0	Q2 (n = 2,860) 2.0–2.75	Q3 (n = 2,816) 2.75–3.74	Q4 (n = 2,818) >3.74	
Sex						<0.001
Male	5,438 (47.49)	926 (29.29)	1,191 (41.89)	1,522 (53.53)	1,799 (64.83)	
Female	5,830 (52.51)	1,848 (70.71)	1,669 (58.11)	1,294 (46.47)	1,019 (35.17)	
Age (years)	49.63 ± 17.82	49.18 ± 19.45	50.44 ± 18.42	49.86 ± 17.49	49.01 ± 15.70	0.009
Race						<0.001
Non-Hispanic White	5,912 (72.92)	1,462 (72.64)	1,524 (74.62)	1,421 (71.17)	1,505 (73.19)	
Non-Hispanic Black	2,101 (10.28)	678 (12.90)	561 (10.39)	499 (10.60)	363 (7.33)	
Mexican American	1,961 (7.77)	354 (5.60)	450 (6.46)	556 (9.02)	601 (9.94)	
Others	1,294 (9.04)	280 (8.86)	325 (8.53)	340 (9.21)	349 (9.54)	
Education level (years)						<0.001
<9	1,179 (5.20)	205 (3.82)	278 (4.52)	326 (5.50)	370 (6.93)	
9–11	4,436 (35.77)	991 (30.25)	1,065 (32.58)	1,151 (38.44)	1,229 (41.68)	
≥12	5,653 (59.03)	1,578 (65.93)	1,517 (62.90)	1,339 (56.05)	1,219 (51.40)	
Marital status						<0.001
Married	7,101 (65.06)	1,584 (60.15)	1,784 (65.17)	1,855 (66.88)	1,878 (67.96)	
Living alone	4,167 (34.94)	1,190 (39.85)	1,076 (34.83)	961 (33.12)	940 (32.04)	
Family income						<0.001
Low	3,215 (19.07)	712 (17.85)	764 (16.37)	801 (18.60)	938 (23.38)	
Medium	4,349 (35.82)	1,059 (35.26)	1,074 (33.84)	1,103 (36.40)	1,113 (37.77)	
High	3,704 (45.11)	1,003 (46.88)	1,022 (49.79)	912 (45.01)	767 (38.86)	
BMI	29.12 ± 6.67	26.52 ± 6.44	28.75 ± 6.55	30.08 ± 6.56	31.08 ± 6.26	<0.001
Physical activity (MET)	624.86 (0.00, 2,640.00)	670.50 (12.13, 2,400.00)	560.00 (0.00, 2,280.00)	630.00 (0.00, 2,880.00)	720.00 (0.00, 3,180.00)	0.003
Dietary cholesterol (mg)	237.50 (153.50, 368.00)	222.00 (143.62, 345.50)	233.00 (148.50, 356.00)	240.50 (158.38, 376.00)	259.00 (165.00, 394.38)	<0.001
Smoke status	5,337 (46.82)	1,203 (43.52)	1,333 (46.21)	1,312 (45.08)	1,489 (52.30)	<0.001
Alcohol status	8,060 (76.27)	1,986 (77.03)	1,996 (75.45)	2,006 (75.99)	2,072 (76.61)	0.774
Hypertension	3,254 (25.05)	767 (21.80)	827 (24.69)	831 (26.10)	829 (27.53)	0.006
Diabetes	1,264 (7.74)	307 (8.03)	317 (7.09)	310 (7.04)	330 (8.77)	0.129
Cancer	1,096 (8.96)	285 (9.00)	281 (9.06)	297 (10.07)	233 (7.76)	0.192
Bowel health						0.167
Chronic constipation	1,103 (9.41)	320 (10.32)	296 (10.29)	250 (8.96)	237 (8.09)	
Chronic diarrhea	981 (7.89)	224 (7.42)	244 (7.80)	239 (7.24)	274 (9.06)	

Abbreviations: Q: quantile; MET: metabolic equivalent; BMI: body mass index.

^a^ Data are presented as unweighted frequencies (weighted percentage) for categorical variables and mean (SD) or median (IQR) for continuous variables.

### 3.2 Logistic regression analysis

Weighted multivariate logistic regression analysis revealed a significant association between elevated NHHR levels and chronic constipation in females, with an adjusted odds ratio (OR) of 1.11 (95% CI: 1.04–1.20, *P* = 0.005). Notably, when NHHR was categorized into quartiles, the third and fourth quartiles demonstrated stronger associations compared to the lowest quartile (*P* = 0.016 and *P* = 0.004, respectively). However, NHHR showed no significant association with chronic diarrhea in female, with an OR of 1.03 (95% CI: 0.95–1.12, *P* = 0.506). Further analysis across NHHR quartiles confirmed this lack of association. Similarly, male participants demonstrated no significant correlation between NHHR and bowel conditions (see Table 2, Model 3).

**TABLE 2:** The association between NHHR and bowel health (weighted).

	Crude		Model 1		Model 2		Model 3	
	OR (95% CI)	*P*-value	OR (95% CI)	*P*-value	OR (95% CI)	*P*-value	OR (95% CI)	*P*-value
Male								
Chronic diarrhea								
NHHR	1.06 (0.95–1.18)	0.268	1.03 (0.91–1.17)	0.634	1.03 (0.91–1.17)	0.632	1.03 (0.91–1.17)	0.62
Q1	1(Ref)		1(Ref)		1(Ref)		1(Ref)	
Q2	1.17 (0.68–2.01)	0.559	1.07 (0.60–1.94)	0.804	1.08 (0.60–1.96)	0.795	1.09 (0.60–1.99)	0.762
Q3	0.95 (0.63–1.44)	0.802	0.84 (0.53–1.34)	0.462	0.86 (0.53–1.39)	0.518	0.85 (0.53–1.35)	0.475
Q4	1.33 (0.92–1.92)	0.121	1.12 (0.72–1.74)	0.595	1.12 (0.72–1.73)	0.615	1.11 (0.72–1.72)	0.614
*P* for Trend		0.267		0.824		0.838		0.87
Chronic constipation								
NHHR	0.94 (0.79–1.11)	0.433	0.97 (0.83–1.13)	0.654	0.96 (0.83–1.12)	0.63	0.96 (0.83–1.12)	0.616
Q1	1(Ref)		1(Ref)		1(Ref)		1(Ref)	
Q2	0.82 (0.49–1.39)	0.455	0.99 (0.57–1.72)	0.973	1.00 (0.58–1.72)	0.993	1.00 (0.58–1.73)	0.991
Q3	0.57 (0.34–0.94)	0.03	0.66 (0.39–1.12)	0.12	0.64 (0.38–1.10)	0.102	0.63 (0.37–1.08)	0.091
Q4	0.75 (0.44–1.27)	0.276	0.86 (0.49–1.53)	0.608	0.85 (0.48–1.52)	0.582	0.85 (0.48–1.51)	0.576
*P* for Trend		0.187		0.385		0.354		0.336
Female								
Chronic diarrhea								
NHHR	1.11 (1.03–1.20)	0.007	1.04 (0.96–1.13)	0.307	1.03 (0.95–1.12)	0.473	1.03 (0.95–1.12)	0.506
Q1	1(Ref)		1(Ref)		1(Ref)		1(Ref)	
Q2	0.81 (0.58–1.13)	0.202	0.71 (0.50–1.02)	0.061	0.70 (0.49–1.00)	0.05	0.71 (0.50–1.02)	0.061
Q3	1.28 (0.91–1.79)	0.147	1.05 (0.75–1.48)	0.767	1.05 (0.74–1.48)	0.777	1.07 (0.76–1.51)	0.692
Q4	1.49 (1.11–2.00)	0.008	1.14 (0.84–1.53)	0.391	1.10 (0.80–1.49)	0.552	1.11 (0.81–1.51)	0.502
*P* for Trend		0.001		0.108		0.169		0.146
Chronic constipation								
NHHR	1.1 (1.03–1.17)	0.006	1.12 (1.04–1.20)	0.003	1.12 (1.04–1.20)	0.004	1.11 (1.04–1.20)	0.005
Q1	1(Ref)		1(Ref)		1(Ref)		1(Ref)	
Q2	1.01 (0.73–1.39)	0.944	1.10 (0.80–1.50)	0.565	1.09 (0.79–1.51)	0.598	1.09 (0.79–1.51)	0.6
Q3	1.36 (1.01–1.82)	0.041	1.50 (1.10–2.04)	0.012	1.49 (1.08–2.04)	0.016	1.48 (1.08–2.03)	0.016
Q4	1.40 (1.07–1.84)	0.016	1.57 (1.18–2.10)	0.003	1.57 (1.17–2.10)	0.004	1.57 (1.17–2.10)	0.004
*P* for Trend		0.003		<0.001		<0.001		0.001

Model 1:adjusted for age, race, education level, marital status, family income, BMI.

Model 2:adjusted for age, race, education level, marital status, family income, BMI, physical activity, dietary cholesterol, smoke status, alcohol status.

Model 3:adjusted for age, race, education level, marital status, family income, BMI, physical activity, dietary cholesterol, smoke status, alcohol status, hypertension, diabetes, cancer.

Abbreviations: NHHR: non-high-density lipoprotein to high-density lipoprotein cholesterol ratio; OR: odd ratio; CI: confidence interval; Q: quantile; Ref: reference.

### 3.3 Nonlinearity assessment

**FIGURE 2:**
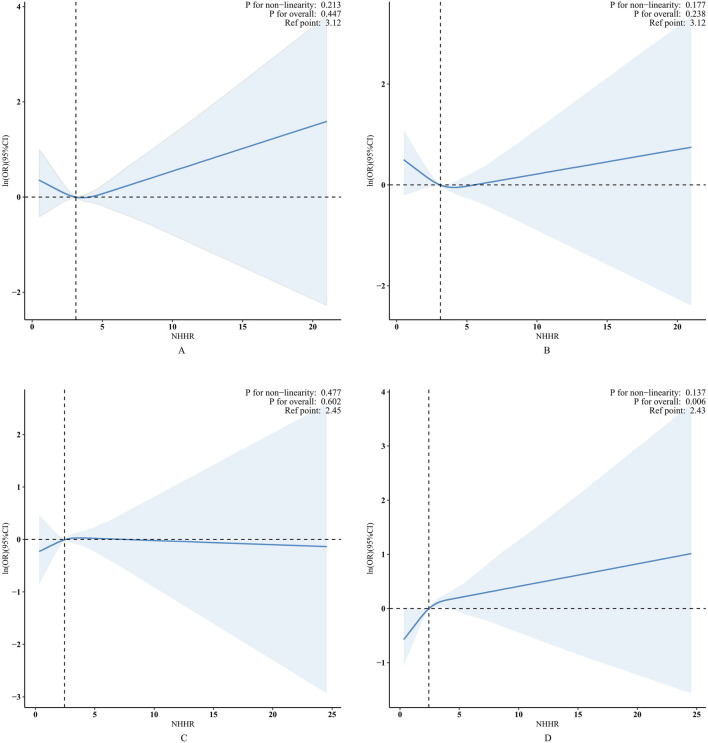
RCS plot of bowel health outcomes by NHHR level. Panels A and B depicting chronic diarrhea and constipation in males, respectively, while Panels C and D represent the same conditions in females. The central thick solid line (blue) indicates the estimated adjusted OR, and the shaded area indicates the 95% CI. The horizontal dashed line (black) indicates that the natural logarithm ln (OR) is 0 and serves as the reference point. The reference point is set to the median level of the NHHR. The vertical dashed line (black) marks the critical value of the NHHR.

Likelihood ratio tests and weighted fitted curves indicated a linear relationship between NHHR and bowel health outcomes, with no significant nonlinear associations detected (females: *P for nonlinearity* = 0.477 and 0.137; males: *P for nonlinearity* = 0.213 and 0.177). The positive association between NHHR and chronic constipation in United States women remained significant after adjusting for potential confounders, indicating an 11% increase in risk per standard unit increase in NHHR (see Figure 2).

### 3.4 Subgroup, sensitivity and additional analyses

**FIGURE 3:**
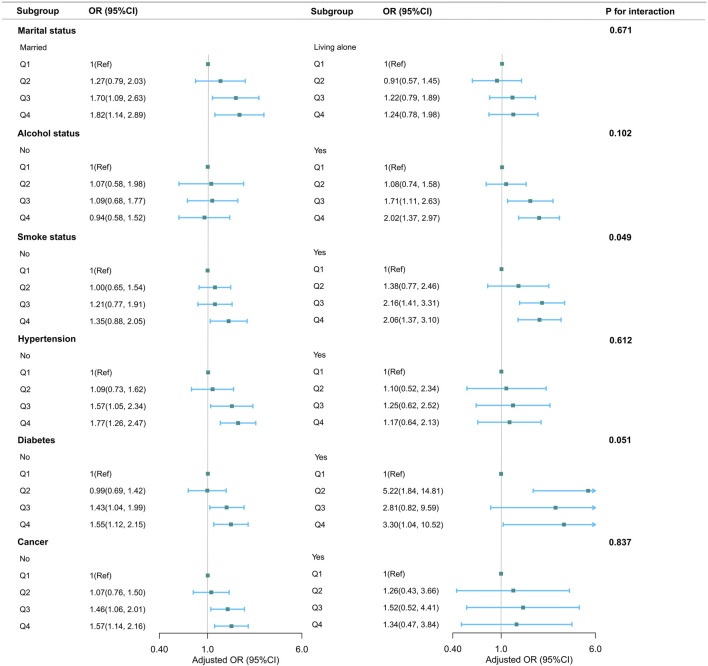
Association between NHHR and bowel health stratified according to basic characteristics. In addition to the stratification components themselves, each stratification factor was adjusted for all other variables (age, race, education level, marital status, family income, BMI, physical activity, dietary cholesterol, smoke status, alcohol status, hypertension, diabetes, cancer).

Subgroup analyses, categorized by marital status, alcohol consumption, smoking habits, hypertension, diabetes, and tumor history, showed generally consistent associations between NHHR and chronic constipation in women across different demographic and lifestyle factors (see Figure 3). Notably, smokers demonstrated a significantly higher likelihood of chronic constipation than nonsmokers, as indicated by a *P*-value for interaction of 0.049. The adjusted OR for chronic constipation, compared to the lowest NHHR quartile, were 1.38, 2.16, and 2.06 for the second, third, and fourth quartiles, respectively. Due to the potential for multiple comparisons, a cautious interpretation of these results is warranted.

Sensitivity analyses, excluding participants with hypercholesterolemia or IBD, similarly confirmed the association between NHHR and chronic constipation. In addition, we performed regression analyses after multiple imputation for missing covariates or included the use of lipid-lowering drugs as a dichotomous variable in the regression model. The results of these analyses were consistent with our primary findings, further verifying the robustness of our conclusions (Supplementary Tables S2-S5).

## 4 Discussion

This study provides novel insights into the correlation between NHHR and bowel health, focusing on chronic diarrhea and constipation within a diverse and representative United States population, an area that has received limited research attention. The study’s findings indicate a significant positive correlation between elevated NHHR levels and the prevalence of chronic constipation among United States females, with no observed association for chronic diarrhea. Conversely, among males, no correlation was observed between NHHR levels and bowel health.

Notably, subgroup analysis revealed a significantly stronger association between NHHR and chronic constipation specifically among female smokers. This suggests a potential interactive effect between smoking behavior, NHHR levels, and bowel health. The stronger association may be due to smoking-induced systemic inflammation (Silveira et al., 2021; Bohlin et al., 2018), gut microbiota alterations (Rebolledo et al., 2017; Yang et al., 2022), and oxidative stress (Caliri et al., 2021), which can worsen constipation and interact with elevated NHHR levels. Hormonal differences in women and the link between smoking and metabolic syndrome may also amplify this effect (Gonenne et al., 2006). However, the precise mechanisms underlying these differences warrant further investigation. Our findings underscore the importance of considering individual differences in clinical practice, essential for developing targeted prevention and treatment strategies for distinct populations and enhancing our comprehension of lipid metabolism’s impact on overall health.

Few studies have directly established an association between abnormal lipid profiles and chronic constipation. While dyslipidemia is frequently linked to unhealthy lifestyle and dietary habits, may also contribute to the development of chronic constipation. For instance, among individuals with type 2 diabetes mellitus and chronic constipation, wheat bran supplementation elevated HDL-C levels and alleviated constipation symptoms (Noureddin et al., 2018). Another study reported a higher prevalence of dyslipidemia among individuals with nonalcoholic steatohepatitis (NASH) and found that functional constipation was associated with NASH in 68.6 percent of cases, suggesting that this comorbidity could intensify the disease trajectory and significantly impair the quality of life for affected individuals (Maevskaya et al., 2015).

Potential biological mechanisms underlying the observed associations include: Firstly, lipids play a crucial role in cell membrane construction and signaling (Kayama and Takeda, 2023). Abnormal lipid metabolism, particularly elevated NHHR levels, is closely linked to chronic low-grade tissue inflammation and oxidative stress, which are central to the development of intestinal disorders (Holtmann et al., 2016). For instance, the Wnt/β-Catenin and PI3K/AKT/mTOR signaling pathways are integral to intestinal cellular stress and inflammatory responses (Izadparast et al., 2022; Moparthi and Koch, 2019). Additionally, pattern recognition receptors like TLR4 are essential for intestinal innate immunity and inflammatory activation (Bruning et al., 2021), and their activation is associated with inflammatory responses induced by dyslipidemia. Moreover, changes in lipid profiles can influence the composition and function of the intestinal microbiota (Rebolledo et al., 2017; Gradisteanu et al., 2022). The intestinal microbiota is vital for maintaining intestinal motility and barrier integrity. The GLP-1 receptor and MAPK signaling pathways are crucial for regulating the effects of the intestinal microbiota on host metabolism. For example, GLP-1 receptor agonists improve glycemic control modulate lipid metabolism and intestinal function by modulating the intestinal microbiota (Batiha et al., 2023). Metabolic bariatric surgery induces significant changes in gastrointestinal hormone secretion, thereby including GLP-1 and PYY, which improve glycemic control and manage metabolic syndrome (Kehagias et al., 2023). These surgeries also alter the gut microbiota, potentially enhancing intestinal function. These findings highlight the therap eutic potential of targeting these pathways and suggest that similar mechanisms may underlie the association between NHHR and bowel health.

The study’s strengths are notable. Firstly, a key strength of our study is the utilization of a large, nationally representative NHANES sample, bolstering the validity and generalizability of our findings. Secondly, rigorous statistical methods, such as weighted analysis and multivariate stepwise logistic regression, were employed. These methodologies effectively mitigated potential confounding variables, yielding precise estimates regarding the relationship between NHHR and bowel health outcomes. Moreover, the robustness of our primary findings was confirmed through sensitivity analyses. This approach not only underscores our meticulous approach in addressing model settings and data source biases but also enhances the reliability and broad applicability of our conclusions across varied scenarios.

Despite these strengths, certain limitations require consideration and suggest directions for future research. The cross-sectional design of the study limits our ability to establish causality. The study did not clarify the direction of the association between NHHR and bowel health, which raises the possibility of reverse causality. Additionally, although a wide range of covariates were included, unmeasured factors may have influenced the results, introducing potential bias. The reliance on self-reported data could also lead to inaccuracies in assessing gut health status. Moreover, the study lacked detailed information on the duration and severity of diarrhea and constipation episodes and may not have fully accounted for the potential impact of lipid-lowering medications on the outcomes. Furthermore, detailed information on previous gastrointestinal surgeries, specific electrolyte levels, medication use causing pseudo-obstruction, thyroid function, and detailed dietary patterns (beyond the 24-h dietary recall) was unavailable in the NHANES dataset for the years we analyzed. These limitations constrained our ability to include these factors in our models. Our study relies on NHANES data, which lacks clinical diagnoses of dyslipidemia or hyperlipidemia. Though our sensitivity analysis shows robust findings, future studies should use datasets with clinical diagnostic criteria to further validate our results. Future prospective, longitudinal studies are needed to address these limitations and provide a more comprehensive understanding.

## 5 Conclusion

In summary, our findings underscore the complex interplay between NHHR and bowel health, particularly in women experiencing constipation, emphasizing the necessity for a more nuanced comprehension of how lipid metabolism influences intestinal function. The potential interactive effects of smoking and other lifestyle factors warrant further exploration. As our research continues, the incorporation of these insights into clinical guidelines may pave the way for more personalized and effective approaches to managing bowel health.

## Data Availability

The raw data supporting the conclusions of this article will be made available by the authors, without undue reservation.

## Abbreviations

RCS, restricted cubic spline; LDL-C, low-density lipoprotein cholesterol; HDL-C, high-density lipoprotein cholesterol; IHD, ischemic heart disease; Non-HDL-C, non-high-density lipoprotein cholesterol; IBS, irritable bowel syndrome; CDC, Centers for Disease Control and Prevention; NCHS, National Center for Health Statistics; BSFS, Bristol Stool Form Scale; MEC, Mobile Examination Centers; TC, total cholesterol; BMI, body mass index; IBD, inflammatory bowel disease; NASH, nonalcoholic steatohepatitis.
