# Supplementary material for: Association between non-high-density lipoprotein to high-density lipoprotein cholesterol ratio and bowel health in U.S. adults: a cross-sectional study
Source: Front Physiol. 2025 Apr 24;16:1501171. doi: 10.3389/fphys.2025.1501171 (PMC12058776; doi:10.3389/fphys.2025.1501171)
Supplement: Supplementary file 1 [file Table1.docx]

**Table S1. Basic characteristics of excluded and included participants.**

| **Characteristic** | **Total**  **(n = 31034)** | **Excluded population**  **(n = 19766)** | **Included population**  **(n = 11268)** |
| --- | --- | --- | --- |
| Age (year) | 31.18 ± 24.93 | 20.67 ± 22.15 | 49.63 ± 17.82 |
| Sex, n (%) |  |  |  |
| Male | 15401 (49.63) | 9963 (50.4) | 5438 (48.26) |
| Female | 15633 (50.37) | 9803 (49.6) | 5830 (51.74) |
| Marital status, n (%) |  |  |  |
| Married | 10365 (55.03) | 3264 (43.14) | 7101 (63.02) |
| Living alone | 8469 (44.97) | 4302 (56.86) | 4167 (36.98) |
| Race/ethnicity, n (%) |  |  |  |
| Non-Hispanic white | 12463 (40.16) | 6551 (33.14) | 5912 (52.47) |
| Non-Hispanic black | 6878 (22.16) | 4777 (24.17) | 2101 (18.65) |
| Mexican American | 7388 (23.81) | 5427 (27.46) | 1961 (17.4) |
| Others | 4305 (13.87) | 3011 (15.23) | 1294 (11.48) |
| Education level (years), n (%) |  |  |  |
| < 9 | 2210 (12.92) | 1031 (17.68) | 1179 (10.46) |
| 9-12 | 6890 (40.29) | 2454 (42.07) | 4436 (39.37) |
| >12 | 8001 (46.79) | 2348 (40.25) | 5653 (50.17) |
| Family income |  |  |  |
| Low | 10666 (37.28) | 7451 (42.97) | 3215 (28.53) |
| Medium | 10575 (36.96) | 6226 (35.9) | 4349 (38.6) |
| High | 7369 (25.76) | 3665 (21.13) | 3704 (32.87) |
| BMI | 25.58 ± 7.64 | 23.08 ± 7.29 | 29.12 ± 6.67 |
| Physical activity (MET) | 560.00 (0.00, 2580.00) | 424.96 (0.00, 2520.00) | 624.86 (0.00, 2640.00) |
| Dietary cholesterol (mg) | 206.00 (128.00, 326.50) | 185.00 (113.50, 297.00) | 237.50 (153.50, 368.00) |
| Smoke status | 8016 (46.82) | 2679 (45.78) | 5337 (47.36) |
| Alcohol status | 10570 (70.92) | 2510 (69.01) | 8060 (71.53) |
| Hypertension | 4889 (25.06) | 1635 (19.84) | 3254 (28.88) |
| Diabetes | 2037 ( 6.89) | 773 (4.22) | 1264 (11.22) |
| Cancer | 1610 ( 9.41) | 514 (8.8) | 1096 (9.73) |
| Bowel health |  |  |  |
| Chronic constipation | 1483 (10.13) | 380 (11.26) | 1103 (9.79) |
| Chronic diarrhea | 1314 ( 8.97) | 333 (9.86) | 981 (8.71) |
| NHHR | 2.45 | 2.31 | 3.15 |

Abbreviations: Q: quantile; MET: metabolic equivalent; BMI: body mass index; NHHR: non-high-density lipoprotein to high-density lipoprotein cholesterol ratio; OR: odd ratio.

**Table S2. The association between NHHR and bowel health after exclusion of hypercholesterolemia (weighted)**

|  | **Crude** | |  | **Model 1** | |  | **Model 2** | |  | **Model 3** | |
| --- | --- | --- | --- | --- | --- | --- | --- | --- | --- | --- | --- |
|  | OR (95% CI) | *P*­Value |  | OR (95% CI) | *P*­Value |  | OR (95% CI) | *P*­Value |  | OR (95% CI) | *P*­Value |
| **Male** |  |  |  |  |  |  |  |  |  |  |  |
| Chronic diarrhea |  |  |  |  |  |  |  |  |  |  |  |
| NHHR | 1.0 (0.81~1.22) | 0.965 |  | 0.91 (0.73~1.14) | 0.404 |  | 0.9 (0.72~1.13) | 0.358 |  | 0.89 (0.71~1.12) | 0.321 |
| Q1 | 1(Ref) |  |  | 1(Ref) |  |  | 1(Ref) |  |  | 1(Ref) |  |
| Q2 | 1.20 (0.65~2.22) | 0.547 |  | 1.12 (0.58~2.17) | 0.727 |  | 1.12 (0.58~2.16) | 0.729 |  | 1.12 (0.59~2.16) | 0.716 |
| Q3 | 0.91 (0.50~1.66) | 0.746 |  | 0.78 (0.40~1.54) | 0.462 |  | 0.78 (0.39~1.54) | 0.461 |  | 0.78 (0.39~1.54) | 0.456 |
| Q4 | 0.92 (0.53~1.63) | 0.781 |  | 0.73 (0.40~1.33) | 0.297 |  | 0.71 (0.39~1.29) | 0.255 |  | 0.70 (0.39~1.27) | 0.234 |
| *P* for Trend |  | 0.49 |  |  | 0.115 |  |  | 0.094 |  |  | 0.085 |
| Chronic constipation |  |  |  |  |  |  |  |  |  |  |  |
| NHHR | 0.8 (0.62~1.03) | 0.076 |  | 0.85 (0.68~1.06) | 0.149 |  | 0.85 (0.68~1.06) | 0.137 |  | 0.84 (0.67~1.05) | 0.124 |
| Q1 | 1(Ref) |  |  | 1(Ref) |  |  | 1(Ref) |  |  | 1(Ref) |  |
| Q2 | 0.82 (0.49~1.39) | 0.265 |  | 0.76 (0.33~1.74) | 0.502 |  | 0.77 (0.33~1.79) | 0.532 |  | 0.76 (0.33~1.75) | 0.508 |
| Q3 | 0.57 (0.34~0.94) | 0.383 |  | 0.94 (0.54~1.63) | 0.814 |  | 0.93 (0.55~1.57) | 0.773 |  | 0.92 (0.53~1.58) | 0.748 |
| Q4 | 0.75(0.44~1.27) | 0.019 |  | 0.58 (0.34~0.99) | 0.046 |  | 0.57 (0.33~0.99) | 0.044 |  | 0.56 (0.33~0.96) | 0.037 |
| *P* for Trend |  | 0.036 |  |  | 0.093 |  |  | 0.07 |  |  | 0.063 |
| **Female** |  |  |  |  |  |  |  |  |  |  |  |
| Chronic diarrhea |  |  |  |  |  |  |  |  |  |  |  |
| NHHR | 1.34 (1.17~1.55) | <0.001 |  | 1.22 (1.06~1.40) | 0.006 |  | 1.2 (1.04~1.38) | 0.013 |  | 1.2 (1.05~1.38) | 0.011 |
| Q1 | 1(Ref) |  |  | 1(Ref) |  |  | 1(Ref) |  |  | 1(Ref) |  |
| Q2 | 0.90 (0.59~1.37) | 0.611 |  | 0.82 (0.52~1.29) | 0.381 |  | 0.80 (0.51~1.24) | 0.308 |  | 0.81 (0.51~1.26) | 0.332 |
| Q3 | 0.93 (0.60~1.44) | 0.732 |  | 0.78 (0.49~1.23) | 0.273 |  | 0.77 (0.48~1.23) | 0.265 |  | 1.77 (0.47~1.25) | 0.274 |
| Q4 | 1.72 (1.21~2.43) | 0.003 |  | 1.32 (0.92~1.87) | 0.132 |  | 1.26 (0.89~1.79) | 0.188 |  | 1.27 (0.90~1.79) | 0.168 |
| *P* for Trend |  | 0.01 |  |  | 0.177 |  |  | 0.234 |  |  | 0.218 |
| Chronic constipation |  |  |  |  |  |  |  |  |  |  |  |
| NHHR | 1.32 (1.16~1.49) | <0.001 |  | 1.37 (1.21~1.54) | <0.001 |  | 1.35 (1.18~1.54) | <0.001 |  | 1.34 (1.17~1.54) | <0.001 |
| Q1 | 1(Ref) |  |  | 1(Ref) |  |  | 1(Ref) |  |  | 1(Ref) |  |
| Q2 | 0.95 (0.67~1.34) | 0.752 |  | 0.98 (0.69~1.39) | 0.9 |  | 0.96 (0.67~1.37) | 0.817 |  | 0.96 (0.67~1.38) | 0.823 |
| Q3 | 1.07 (0.76~1.51) | 0.7 |  | 1.16 (0.83~1.64) | 0.374 |  | 1.16 (0.81~1.64) | 0.409 |  | 1.16 (0.82~1.66) | 0.392 |
| Q4 | 1.61 (1.17~2.20) | 0.004 |  | 1.72 (1.27~2.33) | <0.001 |  | 1.67 (1.20~2.32) | 0.004 |  | 1.66 (1.19~2.31) | 0.004 |
| *P* for Trend |  | 0.005 |  |  | <0.001 |  |  | 0.003 |  |  | 0.004 |

Model 1：adjusted for age, race, education level, marital status, family income, BMI.

Model 2：adjusted for age, race, education level, marital status, family income, BMI, physical activity, dietary cholesterol, smoke status, alcohol status.

Model 3：adjusted for age, race, education level, marital status, family income, BMI, physical activity, dietary cholesterol, smoke status, alcohol status, hypertension, diabetes, cancer.

Abbreviations: NHHR: non-high-density lipoprotein to high-density lipoprotein cholesterol ratio; OR: odd ratio; CI: confidence interval ; Q: quantile; Ref: reference.

**Table S3. The association between NHHR and bowel health after exclusion of IBD (weighted)**

|  | **Crude** | |  | **Model 1** | |  | **Model 2** | |  | **Model 3** | |
| --- | --- | --- | --- | --- | --- | --- | --- | --- | --- | --- | --- |
|  | OR (95% CI) | *P*­Value |  | OR (95% CI) | *P*­Value |  | OR (95% CI) | *P*­Value |  | OR (95% CI) | *P*­Value |
| **Male** |  |  |  |  |  |  |  |  |  |  |  |
| Chronic diarrhea |  |  |  |  |  |  |  |  |  |  |  |
| NHHR | 1.07 (0.96~1.19) | 0.216 |  | 1.03 (0.91~1.18) | 0.591 |  | 1.03 (0.91~1.18) | 0.603 |  | 1.03 (0.91~1.18) | 0.606 |
| Q1 | 1(Ref) |  |  | 1(Ref) |  |  | 1(Ref) |  |  | 1(Ref) |  |
| Q2 | 1.08 (0.61~1.90) | 0.789 |  | 0.99 (0.54~1.83) | 0.976 |  | 1.00 (0.54~1.85) | 0.988 |  | 1.01 (0.54~1.88) | 0.973 |
| Q3 | 0.92 (0.61~1.40) | 0.702 |  | 0.81 (0.51~1.31) | 0.382 |  | 0.83 (0.51~1.35) | 0.439 |  | 0.82 (0.51~1.31) | 0.391 |
| Q4 | 1.33 (0.93~1.92) | 0.117 |  | 1.11 (0.71~1.72) | 0.641 |  | 1.10 (0.71~1.71) | 0.667 |  | 1.10 (0.71~1.70) | 0.653 |
| *P* for Trend |  | 0.206 |  |  | 0.783 |  |  | 0.801 |  |  | 0.824 |
| Chronic constipation |  |  |  |  |  |  |  |  |  |  |  |
| NHHR | 0.94 (0.79~1.11) | 0.431 |  | 0.97 (0.83~1.13) | 0.662 |  | 0.97 (0.83~1.12) | 0.641 |  | 0.97 (0.83~1.12) | 0.627 |
| Q1 | 1(Ref) |  |  | 1(Ref) |  |  | 1(Ref) |  |  | 1(Ref) |  |
| Q2 | 0.82 (0.49~1.38) | 0.445 |  | 0.99 (0.57~1.72) | 0.973 |  | 1.00 (0.58~1.72) | 0.995 |  | 1.00 (0.58~1.73) | 0.993 |
| Q3 | 0.57 (0.34~0.94) | 0.03 |  | 0.66 (0.39~1.13) | 0.128 |  | 0.65 (0.38~1.11) | 0.111 |  | 0.64 (0.37~1.09) | 0.098 |
| Q4 | 0.75 (0.44~1.28) | 0.282 |  | 0.87 (0.49~1.55) | 0.625 |  | 0.86 (0.48~1.54) | 0.601 |  | 0.86 (0.49~1.53) | 0.6 |
| *P* for Trend |  | 0.193 |  |  | 0.405 |  |  | 0.375 |  |  | 0.359 |
| **Female** |  |  |  |  |  |  |  |  |  |  |  |
| Chronic diarrhea |  |  |  |  |  |  |  |  |  |  |  |
| NHHR | 1.12 (1.03~1.20) | 0.005 |  | 1.04 (0.96~1.13) | 0.276 |  | 1.03 (0.95~1.12) | 0.431 |  | 1.03 (0.95~1.12) | 0.46 |
| Q1 | 1(Ref) |  |  | 1(Ref) |  |  | 1(Ref) |  |  | 1(Ref) |  |
| Q2 | 0.80 (0.56~1.13) | 0.201 |  | 0.70 (0.49~1.01) | 0.059 |  | 0.69 (0.48~1.00) | 0.05 |  | 0.70 (0.49~1.02) | 0.06 |
| Q3 | 1.32 (0.93~1.86) | 0.115 |  | 1.08 (0.76~1.52) | 0.673 |  | 1.07 (0.76~1.52) | 0.687 |  | 1.09 (0.77~1.55) | 0.612 |
| Q4 | 1.52 (1.13~2.04) | 0.006 |  | 1.15 (0.85~1.56) | 0.341 |  | 1.11 (0.81~1.52) | 0.489 |  | 1.13 (0.82~1.54) | 0.443 |
| *P* for Trend |  | <0.001 |  |  | 0.078 |  |  | 0.127 |  |  | 0.11 |
| Chronic constipation |  |  |  |  |  |  |  |  |  |  |  |
| NHHR | 1.1 (1.03~1.17) | 0.006 |  | 1.12 (1.04~1.20) | 0.003 |  | 1.12 (1.04~1.20) | 0.004 |  | 1.11 (1.04~1.20) | 0.005 |
| Q1 | 1(Ref) |  |  | 1(Ref) |  |  | 1(Ref) |  |  | 1(Ref) |  |
| Q2 | 1.02 (0.74~1.41) | 0.889 |  | 1.11 (0.80~1.53) | 0.524 |  | 1.10 (0.79~1.53) | 0.552 |  | 1.10 (0.79~1.53) | 0.554 |
| Q3 | 1.38 (1.03~1.84) | 0.034 |  | 1.52 (1.11~2.07) | 0.01 |  | 1.51 (1.09~2.07) | 0.014 |  | 1.50 (1.09~2.06) | 0.014 |
| Q4 | 1.41 (1.07~1.85) | 0.015 |  | 1.58 (1.18~2.11) | 0.003 |  | 1.58 (1.17~2.11) | 0.004 |  | 1.58 (1.17~2.12) | 0.004 |
| *P* for Trend |  | 0.003 |  |  | <0.001 |  |  | <0.001 |  |  | <0.001 |

Model 1：adjusted for age, race, education level, marital status, family income, BMI.

Model 2：adjusted for age, race, education level, marital status, family income, BMI, physical activity, dietary cholesterol, smoke status, alcohol status.

Model 3：adjusted for age, race, education level, marital status, family income, BMI, physical activity, dietary cholesterol, smoke status, alcohol status, hypertension, diabetes, cancer.

Abbreviations: NHHR: non-high-density lipoprotein to high-density lipoprotein cholesterol ratio; OR: odd ratio; CI: confidence interval ; Q: quantile; Ref: reference.

**Table S4. The association between NHHR and bowel health after multiple imputation (weighted)**

|  | **Crude** | |  | **Model 1** | |  | **Model 2** | |  | **Model 3** | |
| --- | --- | --- | --- | --- | --- | --- | --- | --- | --- | --- | --- |
|  | OR (95% CI) | *P*­Value |  | OR (95% CI) | *P*­Value |  | OR (95% CI) | *P*­Value |  | OR (95% CI) | *P*­Value |
| **Male** |  |  |  |  |  |  |  |  |  |  |  |
| Chronic diarrhea |  |  |  |  |  |  |  |  |  |  |  |
| NHHR | 1.08 (0.99~1.18) | 0.068 |  | 1.06 (0.95~1.18) | 0.264 |  | 1.06 (0.95~1.18) | 0.278 |  | 1.06 (0.95~1.18) | 0.271 |
| Q1 | 1(Ref) |  |  | 1(Ref) |  |  | 1(Ref) |  |  | 1(Ref) |  |
| Q2 | 1.20 (0.72~2.00) | 0.48 |  | 1.10 (0.63~1.91) | 0.729 |  | 1.11 (0.63~1.94) | 0.71 |  | 1.13 (0.65~1.98) | 0.66 |
| Q3 | 1.01 (0.70~1.47) | 0.945 |  | 0.91 (0.60~1.36) | 0.628 |  | 0.92 (0.60~1.41) | 0.695 |  | 0.91 (0.60~1.37) | 0.635 |
| Q4 | 1.48 (1.06~2.05) | 0.021 |  | 1.27 (0.85~1.91) | 0.237 |  | 1.26 (0.84~1.90) | 0.253 |  | 1.28 (0.85~1.92) | 0.23 |
| *P* for Trend |  | 0.07 |  |  | 0.372 |  |  | 0.391 |  |  | 0.394 |
| Chronic constipation |  |  |  |  |  |  |  |  |  |  |  |
| NHHR | 0.94 (0.80~1.09) | 0.384 |  | 0.96 (0.83~1.11) | 0.556 |  | 0.95 (0.83~1.10) | 0.506 |  | 0.95 (0.83~1.10) | 0.489 |
| Q1 | 1(Ref) |  |  | 1(Ref) |  |  | 1(Ref) |  |  | 1(Ref) |  |
| Q2 | 0.78 (0.48~1.27) | 0.307 |  | 0.93 (0.56~1.55) | 0.769 |  | 0.93 (0.56~1.55) | 0.781 |  | 0.93 (0.56~1.56) | 0.995 |
| Q3 | 0.58 (0.37~0.91) | 0.018 |  | 0.67 (0.42~1.06) | 0.082 |  | 0.65 (0.41~1.03) | 0.066 |  | 0.64 (0.40~1.01) | 0.056 |
| Q4 | 0.74 (0.45~1.21) | 0.22 |  | 0.83 (0.49~1.42) | 0.487 |  | 0.81 (0.47~1.39) | 0.439 |  | 0.81 (0.47~1.39) | 0.435 |
| *P* for Trend |  | 0.161 |  |  | 0.323 |  |  | 0.278 |  |  | 0.262 |
| **Female** |  |  |  |  |  |  |  |  |  |  |  |
| Chronic diarrhea |  |  |  |  |  |  |  |  |  |  |  |
| NHHR | 1.1 (1.02~1.18) | 0.014 |  | 1.03 (0.95~1.11) | 0.465 |  | 1.02 (0.94~1.11) | 0.634 |  | 1.02 (0.94~1.10) | 0.66 |
| Q1 | 1(Ref) |  |  | 1(Ref) |  |  | 1(Ref) |  |  | 1(Ref) |  |
| Q2 | 0.84 (0.61~1.15) | 0.276 |  | 0.75 (0.54~1.05) | 0.091 |  | 0.74 (0.53~1.04) | 0.077 |  | 0.75 (0.54~1.05) | 0.088 |
| Q3 | 1.28 (0.91~1.82) | 0.156 |  | 1.06 (0.74~1.52) | 0.728 |  | 1.06 (0.74~1.52) | 0.741 |  | 1.08 (0.75~1.55) | 0.676 |
| Q4 | 1.42 (1.07~1.90) | 0.018 |  | 1.10 (0.82~1.47) | 0.528 |  | 1.06 (0.78~1.44) | 0.692 |  | 1.07 (0.79~1.45) | 0.643 |
| *P* for Trend |  | 0.003 |  |  | 0.192 |  |  | 0.269 |  |  | 0.239 |
| Chronic constipation |  |  |  |  |  |  |  |  |  |  |  |
| NHHR | 1.1 (1.04~1.17) | 0.002 |  | 1.13 (1.05~1.21) | 0.001 |  | 1.13 (1.05~1.21) | 0.002 |  | 1.13 (1.05~1.21) | 0.002 |
| Q1 | 1(Ref) |  |  | 1(Ref) |  |  | 1(Ref) |  |  | 1(Ref) |  |
| Q2 | 1.05 (0.78~1.40) | 0.751 |  | 1.13 (0.85~1.50) | 0.388 |  | 1.12 (0.84~1.50) | 0.429 |  | 1.12 (0.84~1.50) | 0.432 |
| Q3 | 1.36 (1.03~1.80) | 0.03 |  | 1.50 (1.11~2.02) | 0.009 |  | 1.49 (1.10~2.03) | 0.012 |  | 1.49 (1.10~2.02) | 0.012 |
| Q4 | 1.44 (1.12~1.86) | 0.005 |  | 1.62 (1.25~2.12) | < 0.001 |  | 1.62 (1.24~2.13) | < 0.001 |  | 1.63 (1.24~2.13) | 0.001 |
| *P* for Trend |  | < 0.001 |  |  | < 0.001 |  |  | < 0.001 |  |  | < 0.001 |

Model 1：age, race, education level, marital status, family income, BMI.

Model 2：age, race, education level, marital status, family income, BMI, physical activity, dietary cholesterol, smoke status, alcohol status.

Model 3：age, race, education level, marital status, family income, BMI, physical activity, dietary cholesterol, smoke status, alcohol status, hypertension, diabetes, cancer.

Abbreviations: NHHR: non-high-density lipoprotein to high-density lipoprotein cholesterol ratio; OR: odd ratio; CI: confidence interval ; Q: quantile; Ref: reference.

**Table S5. Association between NHHR and bowel health after including lipid-lowering medications (weighted)**

|  | **Crude** | |  | **Model 1** | |  | **Model 2** | |  | **Model 3** | |
| --- | --- | --- | --- | --- | --- | --- | --- | --- | --- | --- | --- |
|  | OR (95% CI) | *P*­Value |  | OR (95% CI) | *P*­Value |  | OR (95% CI) | *P*­Value |  | OR (95% CI) | *P*­Value |
| **Male** |  |  |  |  |  |  |  |  |  |  |  |
| Chronic diarrhea |  |  |  |  |  |  |  |  |  |  |  |
| NHHR | 1.03 (0.93~1.13) | 0.589 |  | 1.01 (0.90~1.12) | 0.889 |  | 1.01 (0.90~1.12) | 0.915 |  | 0.98 (0.88~1.11) | 0.787 |
| Q1 | 1(Ref) |  |  | 1(Ref) |  |  | 1(Ref) |  |  | 1(Ref) |  |
| Q2 | 1.07 (0.60~1.91) | 0.819 |  | 1.06 (0.57~1.96) | 0.861 |  | 1.04 (0.56~1.91) | 0.909 |  | 1.04 (0.56~1.93) | 0.894 |
| Q3 | 0.68 (0.39~1.19) | 0.173 |  | 0.65 (0.35~1.21) | 0.164 |  | 0.65 (0.33~1.26) | 0.193 |  | 0.61 (0.31~1.19) | 0.141 |
| Q4 | 1.27 (0.77~2.10) | 0.344 |  | 1.19 (0.66~2.16) | 0.55 |  | 1.16 (0.64~2.11) | 0.619 |  | 1.06 (0.58~1.94) | 0.848 |
| *P* for Trend |  | 0.556 |  |  | 0.792 |  |  | 0.853 |  |  | 0.857 |
| Chronic constipation |  |  |  |  |  |  |  |  |  |  |  |
| NHHR | 0.93 (0.78~1.10) | 0.363 |  | 0.96 (0.82~1.12) | 0.583 |  | 0.96 (0.83~1.11) | 0.537 |  | 0.94 (0.80~1.11) | 0.452 |
| Q1 | 1(Ref) |  |  | 1(Ref) |  |  | 1(Ref) |  |  | 1(Ref) |  |
| Q2 | 0.97 (0.45~2.07) | 0.936 |  | 1.07 (0.50~2.31) | 0.849 |  | 1.07 (0.50~2.31) | 0.849 |  | 1.05 (0.49~2.26) | 0.888 |
| Q3 | 0.54 (0.26~1.12) | 0.097 |  | 0.62 (0.30~1.30) | 0.2 |  | 0.63 (0.30~1.32) | 0.213 |  | 0.57 (0.26~1.25) | 0.156 |
| Q4 | 0.82 (0.41~1.67) | 0.585 |  | 0.97 (0.48~1.96) | 0.927 |  | 0.95 (0.46~1.94) | 0.881 |  | 0.88 (0.41~1.89) | 0.728 |
| *P* for Trend |  | 0.372 |  |  | 0.609 |  |  | 0.578 |  |  | 0.457 |
| **Female** |  |  |  |  |  |  |  |  |  |  |  |
| Chronic diarrhea |  |  |  |  |  |  |  |  |  |  |  |
| NHHR | 1.11 (1.02~1.20) | 0.015 |  | 1.05 (0.96~1.14) | 0.284 |  | 1.04 (0.95~1.13) | 0.421 |  | 1.03 (0.94~1.13) | 0.479 |
| Q1 | 1(Ref) |  |  | 1(Ref) |  |  | 1(Ref) |  |  | 1(Ref) |  |
| Q2 | 0.80 (0.49~1.31) | 0.374 |  | 0.73 (0.45~1.21) | 0.219 |  | 0.73 (0.44~1.21) | 0.204 |  | 0.74 (0.45~1.22) | 0.232 |
| Q3 | 1.23 (0.73~2.06) | 0.424 |  | 1.02 (0.61~1.71) | 0.925 |  | 1.03 (0.62~1.71) | 0.914 |  | 1.05 (0.62~1.78) | 0.839 |
| Q4 | 1.60 (1.13~2.27) | 0.009 |  | 1.26 (0.89~1.78) | 0.188 |  | 1.22 (0.85~1.75) | 0.265 |  | 1.24 (0.86~1.77) | 0.234 |
| *P* for Trend |  | 0.002 |  |  | 0.062 |  |  | 0.089 |  |  | 0.077 |
| Chronic constipation |  |  |  |  |  |  |  |  |  |  |  |
| NHHR | 1.1 (1.02~1.21) | 0.014 |  | 1.11 (1.02~1.22) | 0.02 |  | 1.12 (1.02~1.22) | 0.016 |  | 1.11 (1.02~1.21) | 0.014 |
| Q1 | 1(Ref) |  |  | 1(Ref) |  |  | 1(Ref) |  |  | 1(Ref) |  |
| Q2 | 1.00 (0.67~1.47) | 0.981 |  | 1.06 (0.71~1.58) | 0.766 |  | 1.05 (0.70~1.57) | 0.811 |  | 1.05 (0.70~1.57) | 0.812 |
| Q3 | 1.38 (1.01~1.88) | 0.045 |  | 1.49 (1.07~2.07) | 0.02 |  | 1.49 (1.06~2.09) | 0.022 |  | 1.49 (1.06~2.09) | 0.025 |
| Q4 | 1.53 (1.08~2.16) | 0.017 |  | 1.59 (1.12~2.24) | 0.01 |  | 1.59 (1.13~2.23) | 0.009 |  | 1.58 (1.13~2.22) | 0.009 |
| *P* for Trend |  | 0.004 |  |  | 0.003 |  |  | 0.003 |  |  | 0.003 |

Model 1：age, race, education level, marital status, family income, BMI.

Model 2：age, race, education level, marital status, family income, BMI, physical activity, dietary cholesterol, smoke status, alcohol status.

Model 3：age, race, education level, marital status, family income, BMI, physical activity, dietary cholesterol, smoke status, alcohol status, hypertension, diabetes, cancer.

Abbreviations: NHHR: non-high-density lipoprotein to high-density lipoprotein cholesterol ratio; OR: odd ratio; CI: confidence interval ; Q: quantile; Ref: reference.
